# Frequency of mispackaging of *Prochlorococcus* DNA by cyanophage

**DOI:** 10.1038/s41396-020-00766-0

**Published:** 2020-09-14

**Authors:** Raphaël Laurenceau, Nicolas Raho, Mathieu Forget, Aldo A. Arellano, Sallie W. Chisholm

**Affiliations:** 1 Department of Civil and Environmental Engineering, Massachusetts Institute of Technology, Cambridge, MA USA; 2 Department of Biology, Massachusetts Institute of Technology, Cambridge, MA USA; 3 Present Address: Institut de Biologie de l’Ecole Normale Supérieure, Département de Biologie, Ecole Normale Supérieure, CNRS, INSERM, PSL Research University, Paris, France

**Keywords:** Environmental microbiology, Ecology

## Abstract

*Prochlorococcus* cells are the numerically dominant phototrophs in the open ocean. Cyanophages that infect them are a notable fraction of the total viral population in the euphotic zone, and, as vehicles of horizontal gene transfer, appear to drive their evolution. Here we examine the propensity of three cyanophages—a podovirus, a siphovirus, and a myovirus—to mispackage host DNA in their capsids while infecting *Prochlorococcus*, the first step in phage-mediated horizontal gene transfer. We find the mispackaging frequencies are distinctly different among the three phages. Myoviruses mispackage host DNA at low and seemingly fixed frequencies, while podo- and siphoviruses vary in their mispackaging frequencies by orders of magnitude depending on growth light intensity. We link this difference to the concentration of intracellular reactive oxygen species and protein synthesis rates, both parameters increasing in response to higher light intensity. Based on our findings, we propose a model of mispackaging frequency determined by the imbalance between the production of capsids and the number of phage genome copies during infection: when protein synthesis rate increase to levels that the phage cannot regulate, they lead to an accumulation of empty capsids, in turn triggering more frequent host DNA mispackaging errors.

## Introduction

With an estimated population of 3 × 10^27^ cells on Earth, *Prochlorococcus* is the most numerically abundant phytoplankton species in the global oceans [1]. Distributed throughout the euphotic zone in mid-latitude habitats, *Prochlorococcus* is responsible for fixing an estimated 4 billion tons of carbon each year, playing a central role in ocean food webs. *Prochlorococcus* genomes are highly streamlined, an adaptation for growth in oligotrophic surface waters [2]. Different ocean regions contain hundreds to thousands of *Prochlorococcus* coexisting subpopulations [3, 4]. Horizontal gene transfer (HGT) has had a large impact on *Prochlorococcus* evolution, both in terms of generating genomic variability in genomic islands [5] or in reinforcing genetic similarity in closely related cells [6, 7]. Furthermore, ways of exchanging genetic information among cells are particularly critical for streamlined organisms such as *Prochlorococcus*; its absence could lead to extinction in the face of environmental change [8].

Notably, Larkin et al. revealed that the drivers of *Prochlorococcus* fine-scale genomic diversity are multiple, complex, encompass biotic and abiotic factors, and remain elusive [9]. As shown recently by Arevalo et al. [7], HGT events leading to homologous recombination between chromosomal segments result in cohesive ‘gene-flow units’ (group of recombinogenic cells) in the environment, which align impressively well with phylogenetically defined ecological populations [7]. This critical observation raises the possibility that the frequency at which those HGT events occur in the environment, cumulated over evolutionary timescales, could participate in explaining the current spatial disparity of fine-scale diversity in wild *Prochlorococcus* populations.

Modes of HGT available to *Prochlorococcus* include lipid-bound vesicles, which contain DNA and are known to be abundant in the oceans [10, 11], natural transformation, but only in some LLIV clades that contain the necessary genes for competence [12], and transduction via phage capsids. The latter is one of the canonical modes of HGT in bacteria [13–15] and is thought to occur at significant frequencies in aquatic ecosystems [16–18]. Given the abundance of cyanophages (phages that infect cyanobacteria) that infect *Prochlorococcus* in the oceans (easily reaching 10^5^ to 10^6^ phages mL^−1^) [19, 20], which represent a notable fraction of the total viral population in the euphotic zone [21], it appears that transduction should be occurring. Surprisingly, however, this likely source of major genetic mixing within marine picocyanobacteria has barely been investigated. The only experimental evidence in that direction was performed by Clokie et al., who showed that the *Synechococcus* myovirus S-PM2 is capable of packaging its host chromosomal DNA at low frequency [22]. Thus, we focused this study on exploring and quantifying this phenomenon.

Specialized (reviewed in [23, 24]) and lateral [25] transduction in bacteria are strictly mediated by lysogenic phages, as they enter and leave their host chromosome. Prophages, the hallmarks of lysogeny, however, have not been observed in the hundreds of *Prochlorococcus* isolates or single-cell genomes from the wild [26], suggesting that the vast majority of phages infecting *Prochlorococcus* are strictly lytic. This leaves generalized transduction—the random mispackaging of host DNA into capsids and its subsequent injection and recombination in a recipient cell—as the dominant route of phage-mediated HGT in these bacteria.

**Fig. 1:**
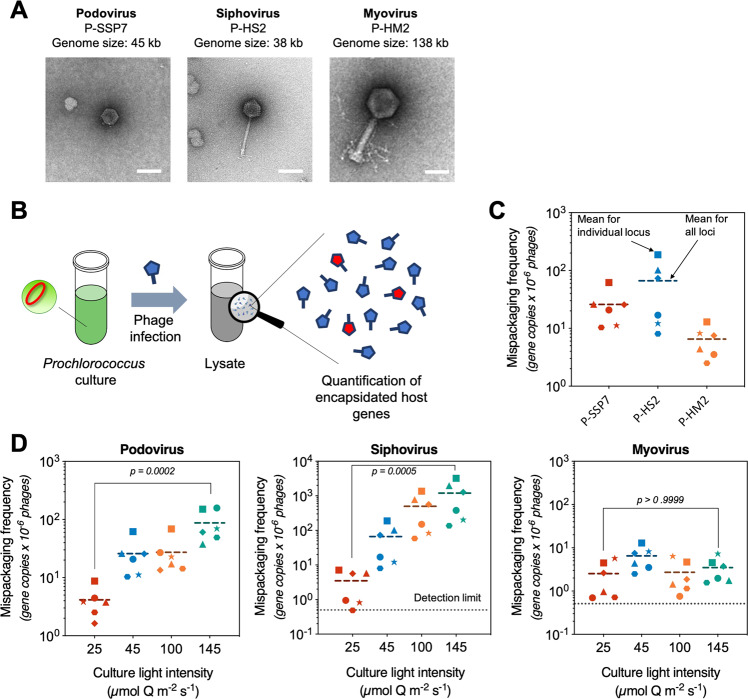
Quantification of mispackaging of host DNA during infection of *Prochlorococcus* MED4 by three cyanophages. **a** Electron micrograph of the three cyanophages infecting *Prochlorococcus* MED4 used in this study. The scale bar is 50 nm. **b** Cartoon illustrating the experimental method. A cyanophage infection typically results in the production of a small fraction of cyanophage capsids having mispackaged host DNA, represented in red. **c** Detection of host DNA mispackaging inside cyanophage capsids under standard laboratory conditions, at a constant light intensity of 45 µmol Q m^−2^ s^−1^ (see ‘Materials and methods’ for details). The frequency of a gene mispackaging during infection is expressed in gene copies per million phages. Each symbol is the mean of three parallel infections for a given locus (Table 1). The colored dotted line is the mean value of all loci and all replicates. The three colors for the different phage are arbitrary—for ease of visualization. **d** Impact of growth light intensity on mispackaging during infection. The data 45 µmol Q m^−2^ s^−1^ are from (**c**), replotted for comparison. The dotted line at 0.5 gene copies per million phages marks the detection limit of the assay. The four colors for the different light intensities are arbitrary—for ease of visualization. The *p* values indicated between the lowest to highest light intensity samples were calculated by a Kruskal–Wallis test followed by a Dunn test. The graphs are replotted in Supplementary Fig. 1 to highlight locus to locus variations.

To explore the potential of *Prochlorococcus* lytic cyanophages to be vectors of generalized transduction, we quantified the mispackaging of fragments of host DNA during infection of *Prochlorococcus* MED4 by three cyanophages with different morphotypes belonging to the sipho- (P-HS2), myo- (P-HM2), and podovirus (P-SSP7) families (Fig. 1a). Our goal was to determine the frequency of mispackaging in the different morphotypes and begin to explore the environmental factors that might influence mispackaging events in the wild.

## Results and discussion

### Host DNA mispackaging frequency in cyanophage capsids

Using six different loci on the *Prochlorococcus* MED4 chromosome (see positions in Table 1), we quantified the level of mispackaging of MED4 DNA in the capsids of the three different cyanophages (Fig. 1a, Table 2) during infection (Fig. 1b, c). The packaging strategy of the podovirus is T7-like [27], the myovirus’ is likely T4-like headful packaging [28] while the siphovirus’ is unknown, but 490 bp direct terminal repeats suggest a T7-like packaging mechanism [29]. Host DNA was detected in the capsids of all three cyanophages (Fig. 1c). The levels of mispackaging are low compared to those reported for high transducing phages in *Staphylococcus aureus* [30] for example, but comparable to levels (~10 gene copies per million phages) reported for the marine *Synechococcus* cyanophage S-PM2 [22].

**Table 1:** List of *Prochlorococcus* MED4 loci used for qPCR quantification of host DNA inside capsids.

Locus #	Symbol on plots	MED4 chromosome position	Length of amplicon (bp)	Corresponding gene	Putative function	Primer sequences (forward and reverse, 5′–3′)
1	★	537,779	216	PMM0569	Dihydroorotase	GGAGGAAAATGCACTTAATCAATTCG TTAACAACTCCCTCTACTCGCC
2	●	773,638 and 1,345,512	94	PMM1398 and PMM0817	High light-inducible protein	GGAAACATGTTTTGTCTTCCGC TACGTAACAACTGGTCAAATAATTCCTGG
3	◆	1,141,081	162	PMM1195	–	GATCGCAAAATAGGTGCAACTATTCTG TTATCCAGAATGGAGTTTTCTTCCAATTG
4	⬢	1,362,140	100	PMM1420	Possible Fumarate reductase subunit	GGCAAATGGCGATTAAATATTAAG CAACTATGCATAGGCAATGC
5	■	1,457,449	150	PMM1515	Site-specific recombinase	GAGTTAGATAATTTAGATAATGAGTGTTTGGGAGAG GCAACCCTCATTACGTAACCTCATCAC
6	▲	1,464,652	164	PMM1524	Photosystem I PsaA protein	CTACCAGGGCCATCACATGG ATCCCCTTAGGAACTGCTGAC

Various loci were initially picked to look for specific gene mispackaging. Overall, our results showed a homogeneous mispackaging across the genome, and the loci for which the primer pairs gave the most robust and reproducible amplification (highest PCR efficiency) were kept for experiments.

**Table 2:** Cyanophages used in this study.

Phage	Morphotype	Host strain	Auxiliary metabolic genes^a^	Packaging strategy^b^	Genome size (kb)	Location of isolation, depth	Date of isolation	Accession number	Genome publication
P-SSP7	Podoviridae	MED4	4	T7-like [27]	45.17	BATS, 100 m	Sep 1999	NC_006882	[81]
P-HS2	Siphoviridae	MED4	0	Likely T7-like	38.12	HOTS, 125 m	Mar 2006	MT490303	[34]
P-HM2	Myoviridae	MED4	11	T4-like [28]	138.32	HOTS, 125 m	Mar 2006	GU075905	[28]

*HOTS* Hawaii Ocean Time Series station, Pacific oligotrophic gyre, *BATS* Bermuda Atlantic Time Series station, Sargasso Sea.

^a^Number of different auxiliary metabolic genes related to either carbon metabolism, photosynthesis, DNA synthesis, or nutrient uptake processes based on references [34, 43].

^b^Packaging strategy classification based on reference [29].

To place these mispackaging frequencies in perspective—podoviruses (T7-like cyanophages) that infect *Prochlorococcus* have been measured at concentration ranging from 3 to 7 × 10^5^ mL^−1^ in a typical seawater sample from their habitat [20]—which means that the frequencies we observed for podovirus P-SSP7 would extrapolate to a potential of up to ~13–30 Mbp of encapsidated host DNA per mL of seawater, enough to encode 13,000–30,000 genes—roughly a 10^−4^ fraction of total *Prochlorococcus* DNA (see calculation in ‘Materials and methods’). Thus, even relatively low frequencies of DNA mispackaging inside capsids can represent a significant amount of *Prochlorococcus* genetic information available for HGT in the global ocean at any one time.

The difference we observe from one cyanophage to another probably reflects the different types of molecular machinery they use for packaging their genome inside capsids; among the three P-HM2’s packaging system appears to be the least prone to mispackaging errors. Of note, phage DNA packaging systems are not necessarily linked to phage morphotype [29]. To the best of our knowledge, all podoviruses infecting marine picocyanobacteria use a T7-like packaging mechanism [31, 32], and although all described cyanomyoviruses are T4-like [28, 33], their higher diversity makes it harder to ascertain that they share the same packaging mechanism. The siphovirus morphotype is by far the one encompassing the greatest overall biological diversity [34–36], and further studies are required to unveil their packaging mechanisms.

### Effect of cell physiology on mispackaging

We next tested if different environmental factors, known to directly affect *Prochlorococcus* physiology, could have an impact on the mispackaging level of the three phages. We first grew the cells at different light intensities, a variable that not only influences the growth rate of *Prochlorococcus* [37] but also influences infection dynamics [38–40]. In fact, many cyanophages carry photosynthesis genes in their genomes to boost light-harvesting during infection [41–43]; like their hosts, cyanophage genes show daylight cycle transcriptional rhythms [38, 44]; and some cyanophages, including the myovirus P-HM2 used in this study, possess light-specific attachment mechanisms to ensure infection during daylight [38, 45].

Increasing growth light intensity significantly increased the mispackaging frequency—by orders of magnitude—for the podo- and siphovirus but did not impact the myovirus (Fig. 1d). The light-induced changes were large compared to the locus to locus variations in mispackaging frequency we observed in the different phages (Supplementary Fig. 1). Because the growth rate of the cultures also increases with light intensity [37] it is difficult to separate the effect of light vs growth rate. Examining the mispackaging frequency as a function of growth rate revealed, however, that growth rate alone cannot explain the results (Supplementary Fig. 2); mispackaging was greatest at the highest light intensity that was slightly inhibitory for growth. Thus, we hypothesized that mispackaging in the podo- and siphovirus might be caused by a physiological property related to the growth light intensity. We tested two such physiological parameters: reactive oxygen species (ROS) toxicity, and the rate of protein synthesis.

### The role of reactive oxygen species in mediating differential levels of mispackaging inside capsids

ROS are abundant in the surface ocean [46–49], and because it lacks the ability to produce catalase, *Prochlorococcus* is highly sensitive and dependent on other species to detoxify them [48, 50–53]. The light intensities used in our experiments did, for the most part, result in an increase in intracellular ROS relative to cells kept in the dark (Supplementary Fig. 3).

**Fig. 2:**
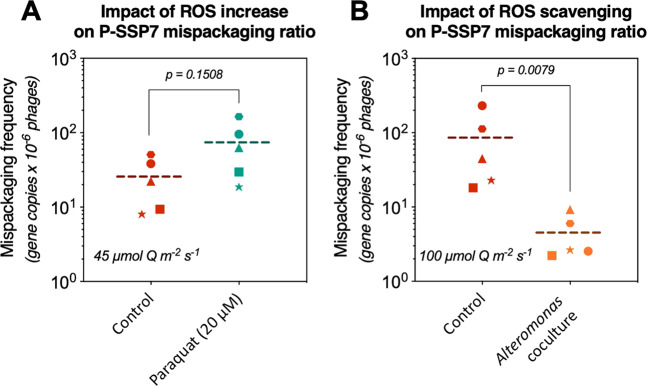
Mispackaging frequency of podovirus P-SSP7 as a function of oxidative stress. **a** Increased oxidative stress (ROS) induced by Paraquat. **b** Decreased oxidative stress induced by the presence of *Alteromonas*, a ‘helper bacteria’ known to reduce oxidative stress when co-cultured with *Prochlorococcus* [50]. The ROS increase experiments were performed at 45 µmol Q m^−2^ s^−1^, to start at a moderate level of ROS inside cells, and the ROS scavenging experiments at 100 µmol Q m^−2^ s^−1^, to compare the effect with a control at an elevated ROS level. The *p* values were calculated in each graph using a Mann–Whitney *U* test.

We thus tested different ways of modulating the ROS toxicity in the culture medium and looked at the impact on mispackaging frequency. To increase intracellular ROS levels, we used the herbicide paraquat, which is known to capture electrons from photosystem I resulting in ROS production [54, 55]. A paraquat treatment at 20 µM at the time of infection triggered an increase in the level of mispackaging inside P-SSP7, but the effect remained below statistical significance (Fig. 2a). To decrease intracellular ROS levels, we tested a coculture of *Prochlorococcus* MED4 grown over many generations with the ‘helper’ heterotrophic *Alteromonas macleodii* strain MIT1002—known to reduce ROS and *Prochlorococcus* oxidative stress via its catalase activity [48, 50, 56]—and compared it with the axenic control. The coculture showed a dramatic decrease in the level of mispacking inside P-SSP7 capsids (Fig. 2b). Taken together, these results did not reject the hypothesis that oxidative stress plays a role in the frequency of mispackaging; more experiments are needed to confirm their role.

Overall, the results suggest that ROS could play a role in mispackaging of host DNA inside cyanophage capsids, helping explain the positive correlation between light intensity and the mispackaging level. That the helper strain, *Alteromonas*, could entirely mitigate the impact of high light intensity on mispackaging frequency was somewhat surprising. While at first glance this makes extrapolating our results to field conditions—where *Prochlorococcus* is surrounded by other bacteria—challenging, there are several things to consider. First, *Prochlorococcus* cells in the upper meter of the surface oceans can experience irradiance levels well above the maximum light level used in this study so the ROS detoxification challenges under those conditions could exceed those encountered in our cultures. Second, copiotrophic bacteria like *Alteromonas* are not the dominant bacteria in *Prochlorococcus’* habitat. And finally, other sources of ROS exist in the wild, such as rainfall or the production by other microbes [52, 57]. Thus although mispackaging could be alleviated by the presence of *Alteromonas* in extremely dense laboratory cultures, this does not mean that mispackaging does not occur in the wild.

### Accumulation of empty capsids during infection may lead to a higher mispackaging frequency

The rate of protein synthesis is another physiological parameter directly influenced by growth light intensity. In the cyanobacterium *Synechocystis* sp. PCC 6803, for example, there is a general upregulation of the translational machinery with increasing light intensity, which keeps increasing even under conditions of photoinhibition, presumably due to increased turnover of proteins subject to photodamage [58]. Moreover, Puxty et al. recently described that increased light levels during infection of a *Synechococcus* myovirus resulted in faster capsid production, while the phage DNA synthesis remained unchanged [40]. Such a decoupling between phage protein and phage DNA synthesis rates, modulated by light intensity, is likely to be a general feature of cyanophages all of which essentially rely on light energy influx to power the infection process [38]. Thus, we hypothesized that if protein translation rates increase to levels that the phage cannot regulate, this could result in an accumulation of empty capsids during infection, increasing the likelihood of host DNA mispackaging events. Indeed, whatever the mechanism by which the host DNA gets loaded inside an empty capsid, the accumulation of latent empty capsids should in turn increase the mispackaging frequency, as long as host DNA is present. Data for marine *Synechococcus* myovirus Syn9 [59, 60] *Prochlorococcus* myovirus P-HM2 [39, 60] and podovirus P-SSP7 [61] show that host DNA degradation happens quickly, within a few hours of infection, but host DNA is never entirely depleted, suggesting that leftover fragments remain available for mispackaging inside capsids until the cell lyses.

Since DNA damage is a consequence of ROS toxicity [49, 62], ROS serves to augment the imbalance between phage protein synthesis (capsid production) and phage DNA synthesis. Kolowrat et al. showed that long-term acclimation of *Prochlorococcus* to chronic high light and UV exposure leads to a delay in chromosome replication, probably caused by DNA lesions and replication fork arrests which slow DNA synthesis [63]. Thus, whether by increasing protein translation rate (effect of higher light intensity), or decreasing the phage DNA replication efficiency (effect of ROS), this would lead to an imbalance between phage DNA replication and capsid production leading to the accumulation of latent empty capsids waiting for phage genome replication.

**Fig. 3:**
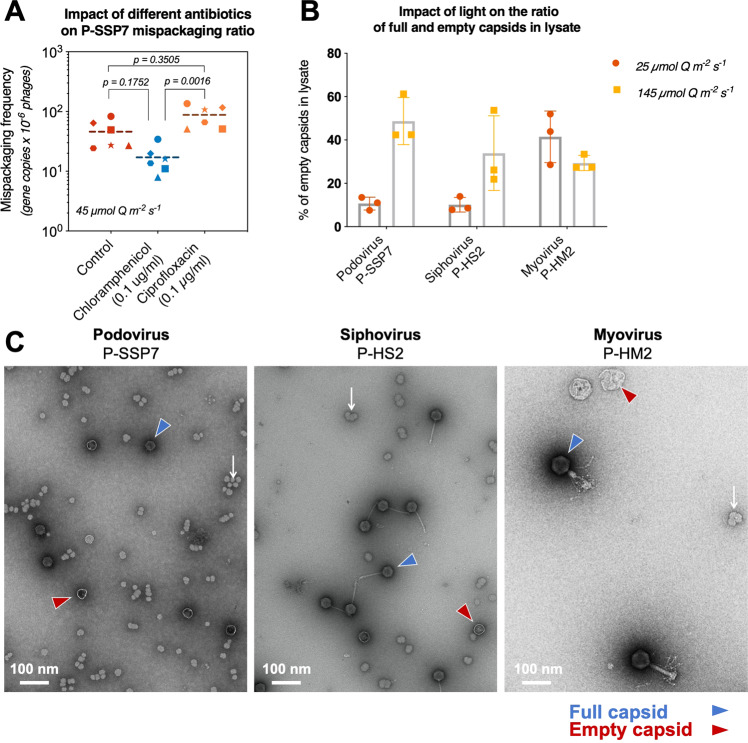
Evaluating the link between mispackaging frequency and the accumulation of empty capsids during infection. **a** Impact of protein (chloramphenicol) or DNA synthesis (ciprofloxacin) inhibition on mispackaging frequency during infection of MED4 by the podovirus. The *p* values were calculated by a Kruskal–Wallis test followed by a Dunn test. The trend of ciprofloxacin and chloramphenicol effect followed our predictions (ciprofloxacin increased the mispackaging frequency, chloramphenicol decreased it), though below statistical significance compared to the control, but their effect was significant when compared to each other. **b** Fraction (%) of empty capsids in the lysate at two light levels. **c** Representative electron micrograph of each cyanophage lysate revealing full (blue arrow) and empty (red arrow) capsids. White arrows show glycogen granules.

We first explored this ‘empty capsid’ hypothesis by inhibiting protein translation and DNA synthesis via the introduction of sublethal doses [64] of the antibiotics chloramphenicol and ciprofloxacin, respectively. Both antibiotics are expected to impact the phage infection but via inhibition of two different host metabolic activities essential for phage particle production: protein translation (chloramphenicol) and DNA synthesis (ciprofloxacin) [65]. We added the antibiotics at the time of infection with the podovirus and observed that, according to our prediction, inhibiting protein translation tended to decrease the frequency of mispackaging while inhibiting DNA synthesis tended to increase it (though the effect was barely significant) (Fig. 3a).

As a more direct way to test the hypothesis, we used negative stain transmission electron microscopy to evaluate the proportion of full and empty capsids from the different treatments (Fig. 3b, c, Supplementary Figs. 4, 5); more specifically, we measured ratios of full to empty capsids during infection by the three phages at two light intensities (Fig. 3b). The fraction of empty capsids rose from ~10 to ~50% for the podovirus with an increase in light intensity—supporting the idea that empty capsids should accumulate at higher light intensities (Fig. 3b). A similar trend was seen in the siphovirus. In contrast, the fraction of empty capsids for the myovirus did not change significantly as a function of light intensity and was roughly 30–40% under both conditions—consistent with lack of light intensity-dependent mispackaging for this phage (Fig. 1d).

The different behavior in response to light of the podo and siphovirus compared to the myovirus—both in terms of mispackaging and full/empty capsid ratios—cannot be explained solely from their different DNA packaging mechanisms (Table 2). It could at least partly be explained by auxiliary metabolic genes (AMGs) encoded in their genome (Table 2). The myovirus P-HM2 genome encodes for 11 different AMGs, including a CP12 homolog, which inhibits the Calvin cycle activity, and a TalC homolog, which reroutes resources to the pentose phosphate pathway for nucleotide production [60], while the podovirus P-SSP7 encodes only four AMGs including TalC, and the siphovirus P-HS2 encodes none (though it possesses other unknown ways to influence the host metabolism) [34]. Thus, the myovirus—better equipped to ‘control’ the host metabolism—might be more resilient in the face of different metabolic regimes imposed by the light level, for example.

To explore this aspect further, we evaluated the three cyanophage’s fitness—as measured by the production of extracellular phage genome copies per infected cell (related to burst size) and the length of the lytic cycle—at two different light levels (Table 3). Indeed, there was reason to suspect that mispackaging of host DNA inside capsids, and the accumulation of empty capsids during infection, might impact overall phage fitness. The results are in accordance with our prediction since the myovirus infection parameters did not vary in function of light, while it changed widely for both the podovirus and siphovirus. Comparing the podovirus and siphovirus patterns, however, we see distinct differences. While the length of the lytic cycle decreased for both under higher light, the production of extracellular phage DNA decreased for the podovirus and increased for the siphovirus. This result was unexpected, given their similar responses to light in terms of host DNA mispackaging and the proportion of empty capsids. This suggests that mispackaging is independent of cyanophage fitness.

**Table 3:** Cyanophage growth parameters in response to host cell growth light intensity, measured by qPCR of phage genome copies in the extracellular and intracellular fraction.

Cyanophage	Podovirus P-SSP7		Siphovirus P-HS2		Myovirus P-HM2	
Light intensity (acclimated host cells)	Low light	High light	Low light	High light	Low light	High light
Extracellular phage genome copies per infected cell^a^	55 ± 7	20 ± 6	40 ± 6	146 ± 40	5 ± 2	5 ± 1
Length of lytic cycle (hours)	24 ± 2	20 ± 2	23 ± 2	17 ± 5	8	8

The extracellular phage genome copies per infected cell and the length of the lytic cycle were calculated for each replicate infection independently, and results correspond to the arithmetic mean of triplicate infection with standard errors. Low light: 25 µmol Q m^−2^ s^−1^, High light: 145 µmol Q m^−2^ s^−1^.

^a^The extracellular phage genome copies per infected cells directly relates to the burst size (the production of infective phage particles per infected cell), but is necessarily higher as it includes noninfective phage particles and phage DNA released during cell lysis.

### Synthesis

**Fig. 4:**
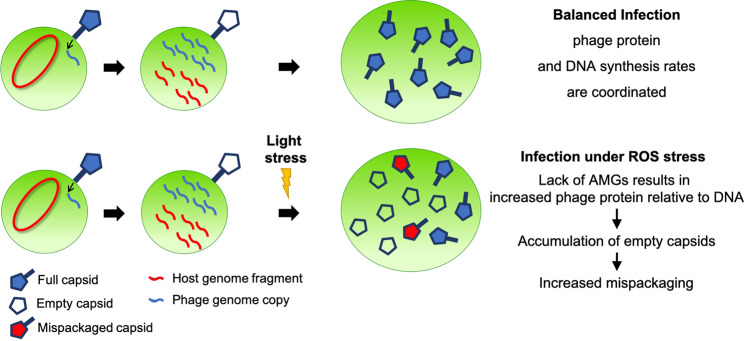
A working model explaining the increased mispackaging of host DNA inside capsids by the podovirus and siphovirus. Higher light levels produce higher intracellular ROS levels and push the host metabolism toward faster protein translation and/or slower DNA synthesis which causes an imbalance in the production rate of capsids and cyanophage genome copies in favor of capsids—a tendency that the myovirus P-HM2 might be able to mitigate thanks to a tighter control of the host metabolism via auxiliary metabolic genes (AMGs) in this cyanophage’s genome. The higher number of latent empty capsids during infection would then increase the likelihood of host DNA mispackaging events.

This is the first report of the encapsidation of *Prochlorococcus* DNA in the capsids of cyanophages. While the myovirus showed a relatively low mispackaging level regardless of conditions, the podo- and siphoviruses showed significant levels of mispackaging at higher light intensities, representing a potential vector for horizontal gene exchange in *Prochlorococcus* by transduction. Our working model proposes that the dependence of mispackaging on light intensity may involve ROS production and/or an imbalance between DNA and protein synthesis during infection (Fig. 4)—a tendency that the myovirus P-HM2 might be able to mitigate thanks to tighter control of the host metabolism via AMGs in its genome.

Interestingly, the underlying mechanisms we describe behind the mispackaging of host DNA inside cyanophage capsids are likely to extend beyond cyanophages. In particular, we observed that mispackaging can be impacted either positively or negatively by antibiotics, a result that could be relevant to the field of phage therapy [66, 67] which is often used in conjunction with antibiotics. In such instances, lateral transfer of DNA through phage capsids could have the unwanted effect of spreading antibiotic resistance genes.

Our results showing an effect of light irradiance on the physiology of infection has potentially significant spaciotemporal implications in the ocean ecosystem: is there greater mispackaging frequency, and production of empty capsids, at the surface compared to deeper in the water column? Are they higher overall in the summer compared to the winter? Are they higher around the equator? Each of these questions deserve dedicated investigations. Higher mispackaging frequencies likely lead locally to a higher flux of HGT, in turn impacting the distribution of picocyanobacteria population diversity [4, 9]. Higher production of empty capsids could impact the dynamics of particulate organic matter, to which phage particles contribute significantly [68].

Many unknowns remain to be studied before we can quantify with any certainty the amount of transduction happening among *Prochlorococcus* cells in the marine environment. For example, only a subset of cyanophage capsids delivers their content, and when they do, multiple barriers exist to prevent the recombination of the incoming exogenous DNA in the recipient cell [13, 14, 69].

Future directions to advance our understanding of transduction in the marine environment will involve the direct measurement of host DNA encapsidation in environmental samples [70–72], or if ways to prepare cyanophage capsids loaded with known and traceable DNA are developed, the direct analysis of transduction events [16].

## Materials and methods

### Culture conditions

Axenic *Prochlorococcus* MED4 cells were grown under constant light flux (45 μmol photons m^−2^ s^−1^ unless otherwise specified) at 24 °C in natural seawater-based Pro99 medium containing 0.2-µm-filtered Sargasso Seawater, amended with Pro99 nutrients (N, P, and trace metals) as previously described [73]. Growth was monitored using bulk culture fluorescence measured with a 10 AU fluorometer (Turner Designs). The presence of heterotroph contaminants was tested as previously described [73], by diluting a small amount of the *Prochlorococcus* culture in three different marine purity test broth. Periodically, the potential presence of contaminants was also examined by flow cytometry with Sybr Green.

For the coculture, *A. macleodii* strain MIT1002 [74] (maintained in ProMM medium—Pro99 medium, as above, plus lactate, pyruvate, glycerol, acetate, and Va vitamins [75]) was spun down and washed twice in Pro99 medium to minimize carryover of trace organic compounds prior to being added to the *Prochlorococcus* MED4 cultures. The coculture was acclimated to constant light flux along with axenic cultures used for comparison.

### Encapsidated DNA extraction

We have set up a protocol to purify *Prochlorococcus* cyanophage-encapsidated DNA free of any host DNA contamination, based on published procedures [22, 30, 70]. Briefly, we omitted the cesium chloride gradient step to optimize the DNA yield per sample, and the potential presence of vesicle-encapsulated DNA was removed by chloroform treatment [11].

In total, 30 mL cultures of exponentially growing *Prochlorococcus* MED4 cells were infected with cyanophage P-SSP7, P-HS2, or P-HM2 at a multiplicity of infection (MOI) ~0.1. The MOI had no effect on the mispackaging frequency (Supplementary Fig. 6). Cultures were preacclimated to the specified light level for several transfers prior to infection. After complete lysis, the lysates were centrifuged at 7000 g for 20 min and filtered on 0.2 µm Steriflip filters (MilliporeSigma, Burlington, MA, USA). Lysates were then concentrated using 15 mL Amicon centrifugal concentrators 100 kDa cutoff (MilliporeSigma) down to a volume of ~2 mL. Samples were treated with 10% volume of chloroform, and washed two times in SM buffer (50 mM Tris pH = 7.5, 100 mM NaCl, 8 mM MgSO_4_) using a 4 mL Amicon centrifugal concentrators 100 kDa cutoff (MilliporeSigma). Of note, the lysate volume was never concentrated below 0.5 mL during washes, as this resulted in significant losses. As a control for digestion, 0.3 µg of pUC19 plasmid DNA (NEB, Ipswich, MA, USA) was added in the sample. The nonencapsidated DNA was then removed by a 1 h incubation at 37 °C with 4 U TURBO DNase enzyme (Thermo-Fisher Scientific), 1X TURBO DNase Buffer, 0.1 mg mL^−1^, RNase A (Thermo-Fisher Scientific) and 1X cOmplete™ EDTA-free Protease Inhibitor Cocktail (Roche, Indianapolis, IN, USA); After 1 h, 4 U TURBO DNase enzyme was added and incubated for another hour at 37 °C. Finally, the encapsidated DNA was extracted using a standard phenol-chloroform extraction, followed by purification on AMPure XP magnetic beads (Beckman Coulter, Brea, CA, USA). Samples were eluted with 50 µL of MilliQ water and used for qPCR quantification.

### Mispackaging quantification by qPCR

DNA samples were quantified using PicoGreen^®^ (Thermo-Fisher Scientific, Waltham, USA) and quantitative PCR reactions were performed with the QuantiTect Probe PCR Kit (Qiagen, Hilden, Germany), using 1 ng of extracted phage DNA in 25 µL reaction volume. Primers for the six amplicons from the *Prochlorococcus* MED4 genome used in all experimental conditions to evaluate mispackaging frequency are listed in Table 1. Amplification was carried out in a CFX96 thermocycler (Bio-Rad, Hercules, CA, USA), cycling conditions were 15 min at 95 °C, followed by 40 cycles of denaturing for 15 s at 94 °C and annealing for 30 s at 55 °C. In each run, the DNA sample was compared to a serial dilution of MED4 genomic DNA from 1 × 10^−9^ g to 1 × 10^−12^ g. The number of encapsidated gene copies was calculated with the formula:

$${\mathrm{GCN}} = \left( {{C} \times {V} \times 10^{ - 9} \times \mathrm{Na} \times {k}} \right)/{M}.$$

GCN: gene copy number in the PCR reaction; *C*: measured concentration from standard curve (ng/µL); *V*: volume of PCR reaction (µL); *M*: molecular weight of host chromosome (g/mol); Na: Avogadro’s number; *k*: number of gene repeats in the genome. The results are then expressed in gene copies per million phages, by dividing the GCN value with the number cyanophage genome copies inside 1 ng of cyanophage DNA, times 1 million. According to qPCR control reactions containing no input template, values below 0.5 gene copies per million phages could be noise (set as the detection limit).

To confirm the complete removal of nonencapsidated DNA, the same qPCR reaction was performed using primers M13F (gtaaaacgacggccagt) and M13R (caggaaacagctatgac) on each sample. If traces of the pUC19 plasmids were detected, samples were considered contaminated and discarded.

### Extrapolation to the natural environment

To extrapolate our results for the podovirus to the natural environment we began with the following assumptions:

1. The value obtained with P-SSP7 applies to all *Prochlorococcus*-infecting podoviruses, measured at concentrations ranging from 300,000 to 700,000 per mL of seawater [20].
2. ‘Mispackaged’ cyanophage capsids are filled with host DNA to capacity, that is ~45 kb, the average podovirus genome size [31].
3. The entire *Prochlorococcus* chromosome can be mispackaged at the mean value we obtained for the 6 loci we tested. So considering the mean value of 25.97 gene copies per million phages at 45 µmol Q m^−2^ s^−1^ (a conservative value, given that under laboratory conditions, mispackaging reached an average of 87.75 gene copies per million phages at 145 µmol Q m^−2^ s^−1^), we extrapolate a mispackaging frequency of 25.97 chromosome copies per million phage capsids in the environment.

A million phage capsids contain 45 kb × 10^6^ = 45,000 Mbp of DNA so that given the assumptions, 25.97 chromosome copies per million phage capsids results in a ratio of phage:host encapsidated DNA of 45,000 Mbp/(25.97 × (*Prochlorococcus* genome size)) = 1045. In other words, 1 out of 1045 capsids is filled with host DNA. Considering 300,000 capsids per mL of seawater, that equates to ~13 Mbp of host encapsidated DNA per mL of seawater. Considering 700,000 capsids, it goes up to ~30 Mbp.

Obviously, this estimation is subject to vast uncertainty, but it serves as an indication of how mispackaging values in gene copies per million phages can relate to the context of *Prochlorococcus* and its phages in the wild.

### Measurement of intracellular ROS

Intracellular ROS levels were measured via H_2_DCFDA assay (Thermo-Fisher Scientific), a cell-permeant that is oxidized to the fluorescent DCF form in the presence of ROS. Fluorescence per cell was measured on an Influx flow cytometer (Becton Dickinson, Franklin Lakes, NJ, USA); cells were excited with a blue 488 nm laser and analyzed for chlorophyll fluorescence (692/40 nm) and DCF (530/40 nm). Calculations of relative fluorescence per cell were done by normalizing red chlorophyll fluorescence per cell to 2 µm reference fluorescent beads (catalog no. 18604; Polysciences, Warrington, PA, USA) as previously described [76]. All flow cytometry data were analyzed using FlowJo version 10.6.1 (FloJo, LLC, Ashland, OR, USA). Triplicate *Prochlorococcus* MED4 cultures acclimated to the different light levels were harvested by centrifugation and resuspended in fresh Pro99 medium before the addition of reconstituted H2DCFDA to a final concentration of 30 µM. Cells were incubated in the dark at room temperature for 30 min, then exposed to their growth light intensity for 30 min, and samples were immediately run on the flow cytometer.

### One-step method for cyanophages growth curve experiment

Infection dynamics were assayed using qPCR to enumerate both intracellular and extracellular cyanophage GCN [60, 61]. Triplicate mid-exponential cultures acclimated to high light (145 μmol Q m^−2^ s^−1^) and low light (50 μmol Q m^−2^ s^−1^) were infected at MOI ~1. To ensure the same MOI was used for a given phage at the two light levels, cell abundances were measured prior to infection and the addition of the cyanophage stock was adjusted accordingly. After 1 h, cultures were diluted 1:10 in fresh Pro99 medium, to stop further infection. Samples were taken immediately after dilution of the infected cultures and then at 2, 5, 8, 11, 14, 17, 20, 23, 26, and 31 h. In total, 200 µL samples were aliquoted into 96-well MultiScreen-HTS GV filter plate with 0.22 µm filters (MilliporeSigma, MSGVN2210) and filtered using a MultiScreen-HTS Vacuum Manifold (MilliporeSigma) into 96-well polystyrene microplates. Filtrates were used for qPCR quantification of extracellular cyanophage genome copies, while filters were used for qPCR quantification of intracellular cyanophage genome copies. Quantitative PCR conditions were the same as above, using primers gaacacttccgcccttacct and ctgcaacgaaagggaattgt for P-SSP7; cgtagagaaggtggcagagg and gaccttccgatgttaaattgc for P-HM2; gaattgctccaatcgtcgtt and cagctcgtgaaaacatcgaa for P-HS2.

In each biological replicate, the burst size was calculated as the total number of cyanophages produced during the lytic cycle (extracellular cyanophage GCN at the end of the lytic cycle minus extracellular cyanophage GCN at *t*_0_) divided by the number of infected cells at *t*_0_ (intracellular cyanophage GCN at *t*_0_). Note that cyanophage latent period lengths and burst sizes are difficult to establish and subject to significant uncertainty. Both parameters are better regarded as relative measures that are specific to the methods and conditions we used to obtain them.

### Quantification of full and empty capsids fractions in cyanophage lysates by electron microscopy

Triplicate mid-exponential cultures acclimated to high light (145 μmol Q m^−2^ s^−1^) and low light (25 μmol Q m^−2^ s^−1^) were infected at MOI ~0.1. Once lysis of the culture was completed, samples were centrifuged at 7000 g for 20 min to remove bacterial debris and filtered onto 0.2 µm Steriflip filter units (MilliporeSigma). In total, 4 mL of each clarified lysate was subsequently ultracentrifuged at 32,000 RPM for 1 h, 4 °C in SW 60 Ti rotor (Beckman Coulter) to pellet cyanophage particles, which were resuspended in 50 µL of fresh and filtered Pro99 medium. In total, 10 μL drops of each suspension were then placed directly on glow discharged carbon-coated grids (EMS, USA) for 1 min. The grids were then blot-dried on filter paper, washed on a drop of ultrapure water, and negatively stained with 2% uranyl acetate in water. Specimens were examined on an FEI Tecnai T12 electron microscope operating at 80 kV at nominal magnifications of 18,500–48,000 and 1–3 μm defocus. Of note, P-HS2 particles were significantly aggregated after resuspension. To allow counting of this phage, Triton X-100 detergent (MilliporeSigma) was added to a final concentration of 0.1% for 1 h at room temperature before grid preparation.

The morphology of empty capsids (Fig. 3c, Supplementary Fig. 4) was consistent with examples from the literature [77–80], and could easily be differentiated from extracellular membrane vesicles (Supplementary Fig. 5). Empty capsids for the tailed cyanophages P-HM2 and P-HS2 were not tailed, consistent with the fact that they are immature capsids that have not been filled, and not capsids that delivered their content inside cells. A minimum of 100 particles (more frequently 200–400 particles) was counted on electron micrographs for each biological replicate to assess the full:empty capsid ratio in the lysate.

## Supplementary information

Supplementary text

Supplementary Figure 1

Supplementary Figure 2

Supplementary Figure 3

Supplementary Figure 4

Supplementary Figure 5

Supplementary Figure 6
