# Supplementary figures and images for: Frequency of mispackaging of Prochlorococcus DNA by cyanophage
Source: ISME J. 2020 Sep 14;15(1):129–40. doi: 10.1038/s41396-020-00766-0 (PMC7852597; doi:10.1038/s41396-020-00766-0)

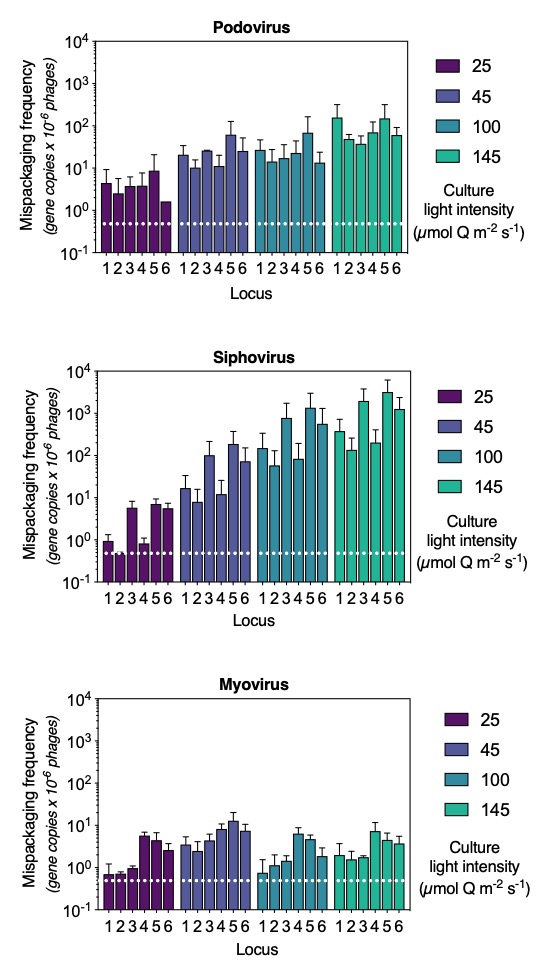

Supplement: Supplementary file 2 — Supplementary Figure 1 [file 41396_2020_766_MOESM2_ESM.jpg]

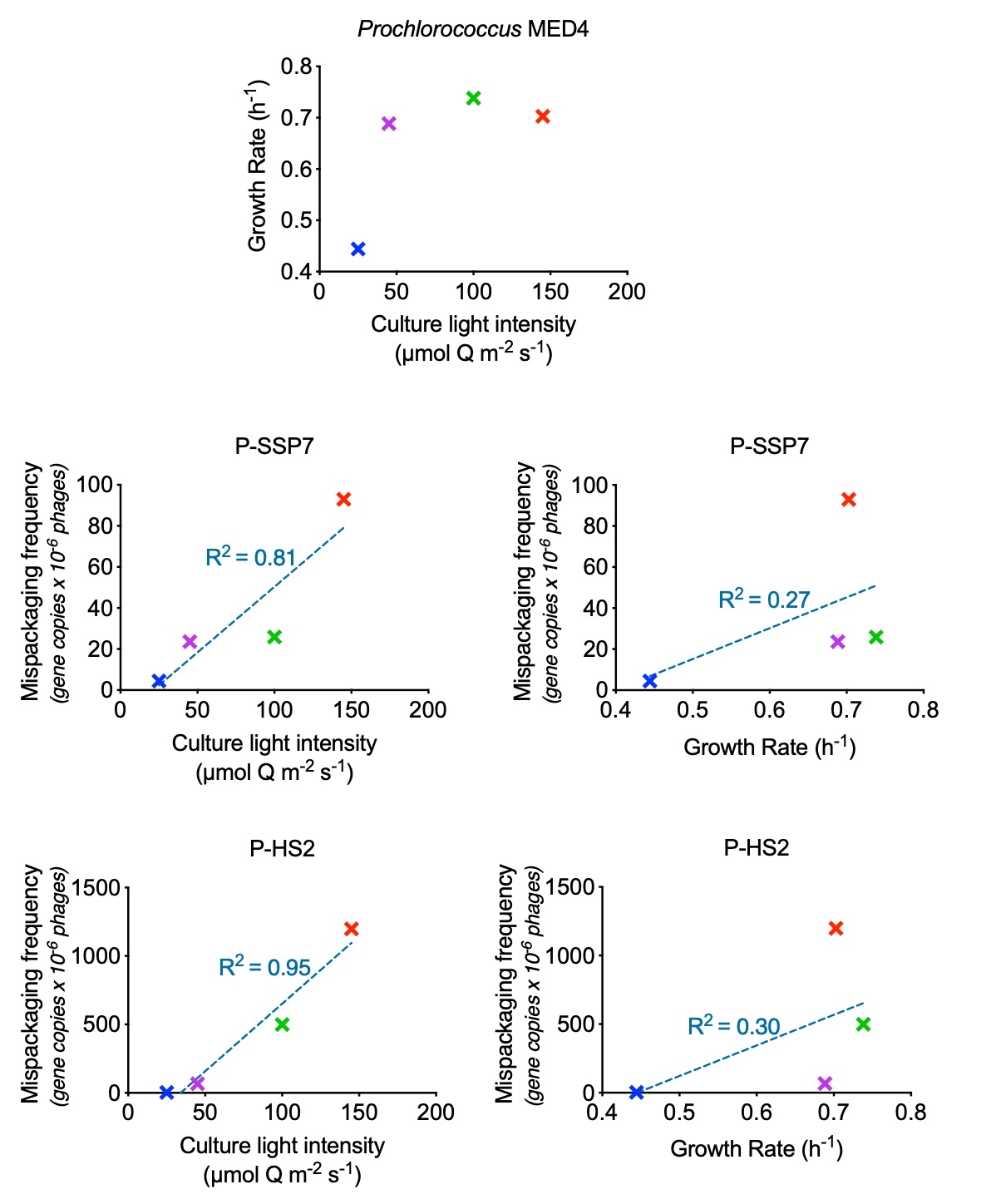

Supplement: Supplementary file 3 — Supplementary Figure 2 [file 41396_2020_766_MOESM3_ESM.jpg]

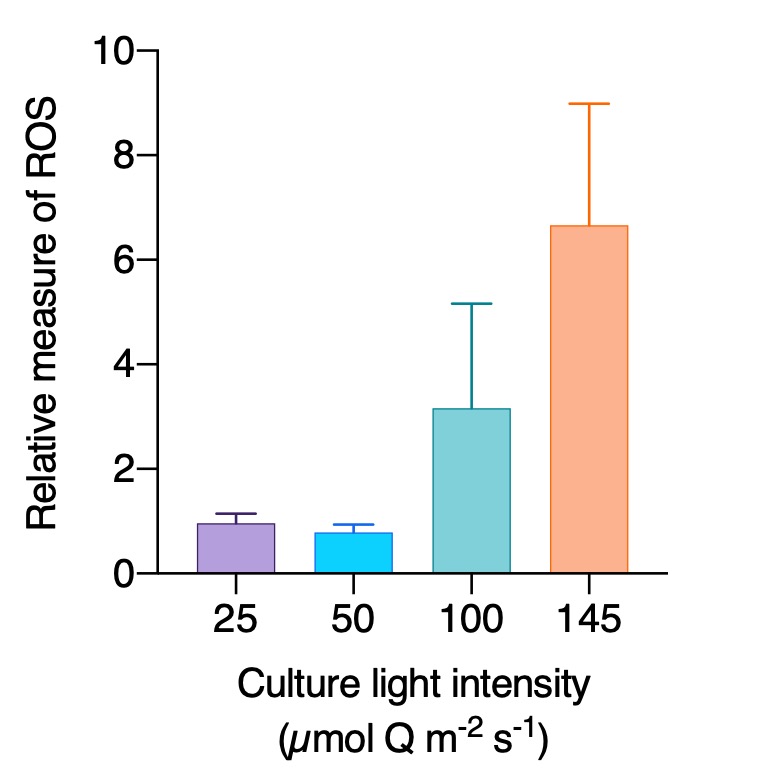

Supplement: Supplementary file 4 — Supplementary Figure 3 [file 41396_2020_766_MOESM4_ESM.jpg]

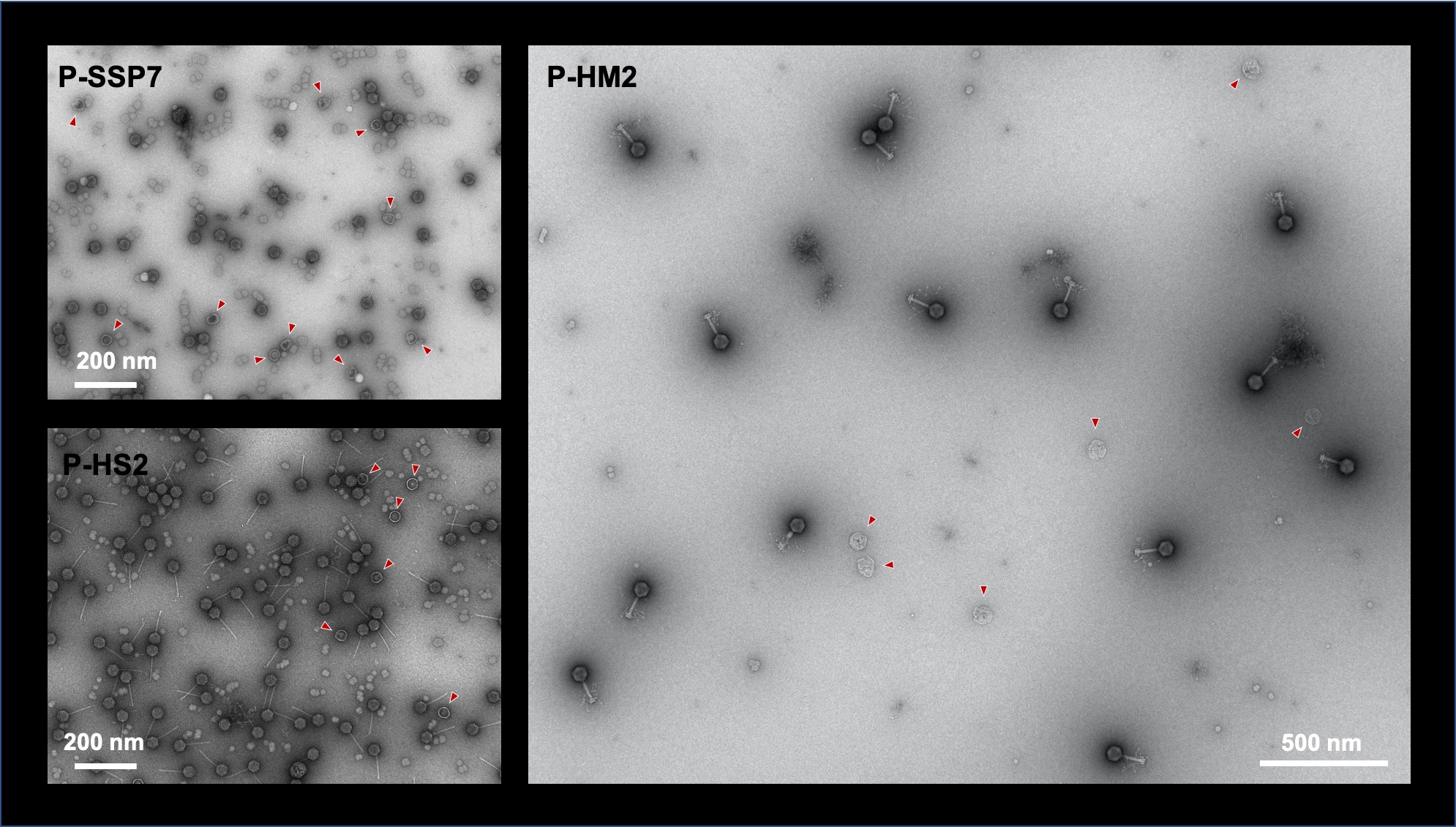

Supplement: Supplementary file 5 — Supplementary Figure 4 [file 41396_2020_766_MOESM5_ESM.jpg]

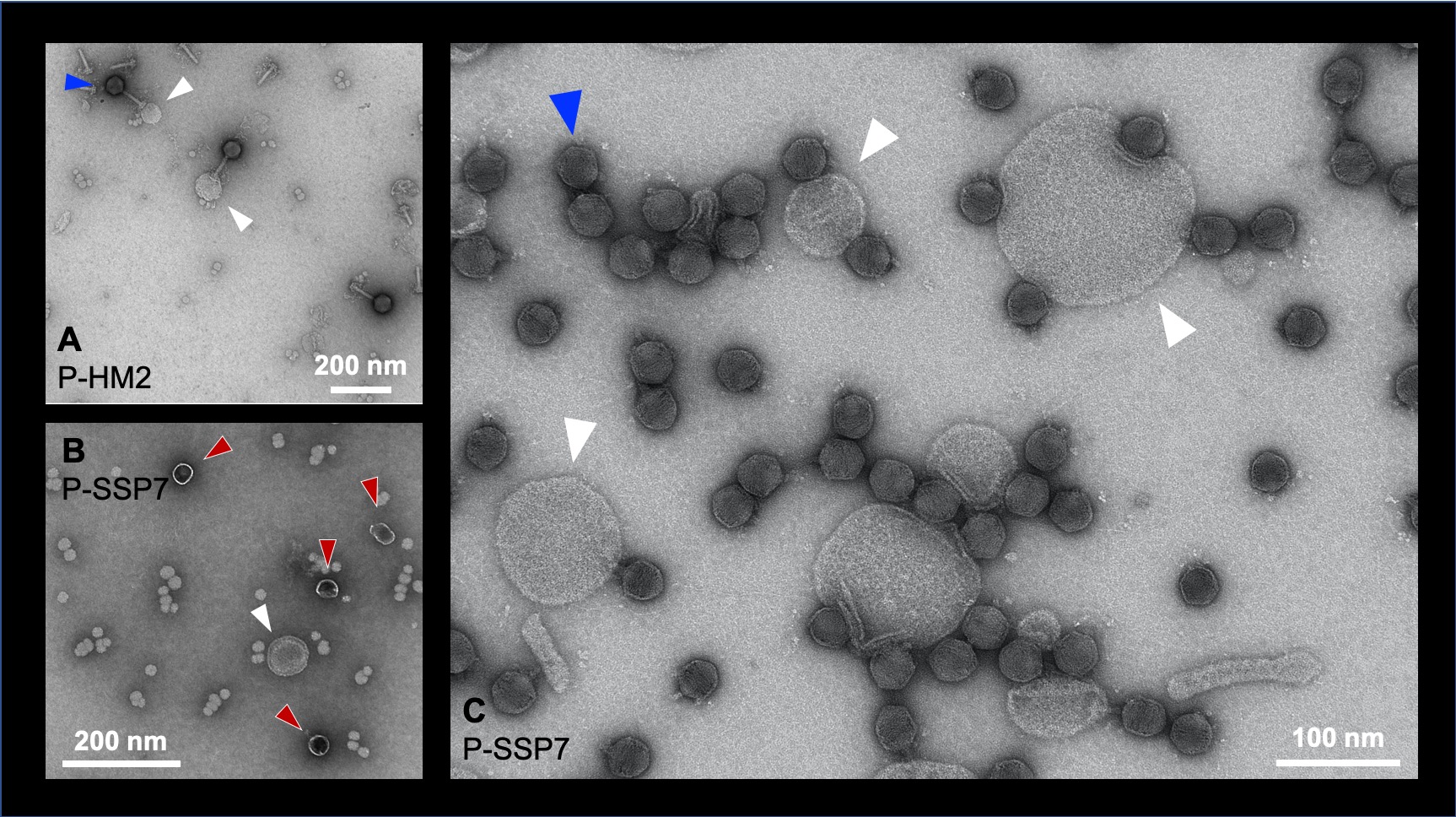

Supplement: Supplementary file 6 — Supplementary Figure 5 [file 41396_2020_766_MOESM6_ESM.jpg]

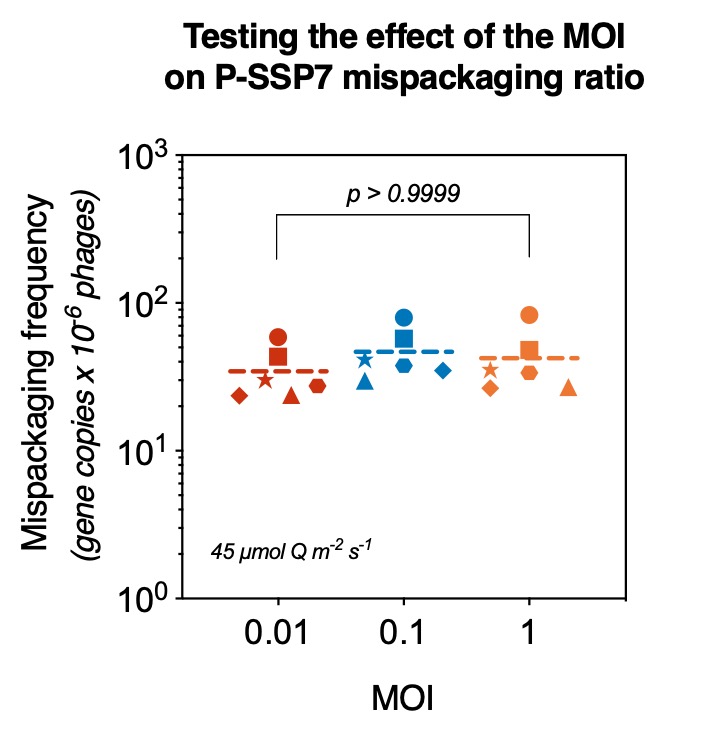

Supplement: Supplementary file 7 — Supplementary Figure 6 [file 41396_2020_766_MOESM7_ESM.jpg]
